# HIF-2α promotes the formation of vasculogenic mimicry in pancreatic cancer by regulating the binding of Twist1 to the VE-cadherin promoter

**DOI:** 10.18632/oncotarget.17999

**Published:** 2017-05-18

**Authors:** Jian Yang, Dong-Ming Zhu, Xiao-Gang Zhou, Ni Yin, Yi Zhang, Zi-Xiang Zhang, De-Chun Li, Jian Zhou

**Affiliations:** ^1^ Department of General Surgery, The First Affiliated Hospital of Soochow University, Suzhou, Jiangsu 215006, China; ^2^ Pancreatic Disease Research Center, The First Affiliated Hospital of Soochow University, Suzhou, Jiangsu 215006, China; ^3^ Department of Oncology, The First Affiliated Hospital of Soochow University, Suzhou, Jiangsu 215006, China

**Keywords:** vasculogenic mimicry (VM), HIF-2α, Twist, VE-cadherin, pancreatic cancer

## Abstract

Vasculogenic mimicry (VM) is a blood supply modality that occurs independently of endothelial cell angiogenesis. Hypoxia and the epithelial-mesenchymal transition (EMT) induce VM formation by remodeling the extracellular matrix. Our previous study demonstrated that hypoxia-inducible factor-2 alpha (HIF-2α) promotes the progress of EMT in pancreatic cancer; however, whether HIF-2α promotes VM formation in pancreatic cancer remains unknown. In this study, we investigated HIF-2α expression and VM by immunohistochemistry in 70 pancreatic cancer patients as well as the role of Twist1and Twist2 in HIF-2α-induced VM *in vitro* and *in vivo*. We found that the overexpression of HIF-2α and VM were correlated with poor tumor differentiation, late clinical stage and lymph node metastasis, and a poor prognosis in pancreatic cancer. Moreover, the upregulation of HIF-2α in SW1990 cells induced VM formation, whereas the opposite results were found after silencing HIF-2α in AsPC-1 cells. A mechanistic study indicated that HIF-2α might regulate the binding of twist1 to vascular endothelial cadherin (VE-cadherin) to promote VM formation in pancreatic cancer cells, and that the P1 (-421bp) and P4 (-2110bp) regions of the Twist1 binding sequences are positive regulatory elements for VE-cadherin. In addition, we confirmed that the overexpression of HIF-2α increased Twist1 expression and promoted tumor growth and VM formation in pancreatic cancer xenografts in nude mice. These findings indicated that HIF-2α might play a critical role in VM and that HIF-2α and the pathway of HIF-2α inducing VM formation are potential therapeutic targets for pancreatic cancer.

## INTRODUCTION

Pancreatic cancer, one of the most lethal cancers among all malignances, is characterized by aggressive local invasion and metastatic spread, with a median overall survival of less than 1year and a 5-year survival of approximately 5% [1]. There are no obvious symptoms in the early stage of pancreatic cancer, and more than half of all pancreatic cancer patients are diagnosed after metastases have occurred, when the chance for surgical resection has been lost [2]. Pancreatic cancer is a complex, multifactorial disease involving mutations in cancer-associated genes, and has a poor prognosis because traditional radiation or chemotherapy strategies yield disappointing results [1, 3]. Thus, it is necessary to understand the molecular pathogenesis of pancreatic cancer, and identify new molecular targets.

Tumor growth and metastasis depend on tumor angiogenesis, a type of neovascularization that occurs in embryonic development and is conventionally regarded as the only way to provide tumor blood supply [4–6]. Anti-angiogenesis drugs that are specific to endothelial cells are a traditional therapy for tumors; however, anti-angiogenesis drugs yield little clinical effect for some malignant tumors. Maniotis *et al*. [7] described an angiogenesis-independent pathway called “Vasculogenic Mimicry (VM),” which is a newly-defined pattern of tumor blood supply in which highly aggressive tumor cells mimic endothelial cells and form extracellular matrix (ECM)-rich structures to supply red cells and plasma without the appearance of endothelial cells. VM channels are surrounded by tumor cells and extracellular matrix, which stain periodic acid-Schiff (PAS) positive, and endothelial cells or makers are absent [8]. The name of VM was created to describe these channels of tumor cells: vasculogenic refers to the channels which contain red blood cells are not come from preexisting vessls; and mimicry indicates that the channels mimic blood vessels but not true blood vessels. Since its discovery, VM has been detected in many other malignant tumors, including hepatocellular carcinoma [9, 10], gallbladder cancer [11], lung cancer [12], prostate cancer [13],and gastric cancer [14]. In fact, VM formation is a multistep and complex process, including activation and proliferation of tumor cells, differentiation of tumor cells into endothelial-like cells and appearance of new vessels. VM has also been linked to increased tumor metastasis rates, disappointing treatment outcomes, and a poor prognosis [15, 16]. Some studies have showed that blood vessels are a three-stage phenomenon containing VM patterns, mosaic blood vessels, and endothelium-dependent vessels [17]. Following tumor growth, VM occurs first, whereas endothelium-dependent vessels ultimately provide the tumor with sufficient blood and promote cancer progression [18]. In various carcinomas, several key proteins are necessary for the formation of VM, among which VE-cadherin is the most critical for VM channels [19, 20].

Along with tumor growth, hypoxia is a characteristic of most solid tumors. Hypoxia-inducing factors (HIF) are involved in VM, angiogenesis, cellular differentiation, tumor progression, chemoresistance, apoptosis, and glucose metabolism, all of which are correlated with a poor prognosis in cancer patients [21]. Hypoxia is involved in angiogenesis and VM formation via many signaling pathways, including the direct modulation of *VEGF-A*, *VEGFR1*, *EPHA2*, *TWIST*, *COX-2*, and *NODAL* expression [22]. Hypoxia inhibits the degradation of HIF, providing a chance for the nuclear localization of HIF-1α and HIF-2α. Previously, HIF-2α has been shown to play an important role in many aspects of digestive cancers, including proliferation, angiogenesis, metastasis, and resistance to chemotherapy [21]. Our previous study indicated that HIF-2α is a vital factor during hypoxia and participates in the epithelial-mesenchymal transition (EMT) in pancreatic cancer [23]; however, there is limited evidence of the role and mechanisms of HIF-2α during VM formation in pancreatic cancer.

In this study, we first investigated the expression and clinical significance of HIF-2α and VM in pancreatic cancer. Next, we studied the role of HIF-2α in the formation of VM in pancreatic cancer cells *in vitro* and *in vivo*. Last, we further investigated the possible mechanism between HIF-2α and VM during the progression of pancreatic cancer.

## RESULTS

### Overexpression of HIF-2αin pancreatic cancer predicts a poor prognosis

**Figure 1:**
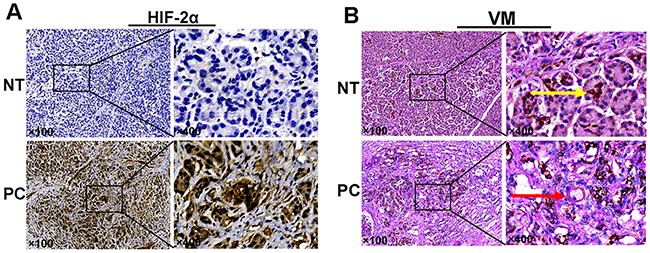
**(A)** Expression of HIF-2α (by IHC). **(B)** VM structure by CD34/PAS double-staining: the yellow arrow indicates endothelial-dependent vessels that are CD34-positive and PAS-positive; the red arrow indicates VM structures that wereCD34-negative and PAS-positive in pancreatic cancer and adjacent non-tumor tissues. Original magnification100× or 400×. The images are representative of three independent experiments. NT: normal tissue; PC: pancreatic cancer.

To detect the effects of HIF-2αon the development of pancreatic cancer, 70 pancreatic cancer tissues and matching adjacent non-tumor tissues were analyzed to investigate the expression of HIF-2α by IHC staining. Based on the results obtained, the localization of HIF-2α was primarily in the cytoplasm and nucleus (Figure 1A). As well, HIF-2α was expressed in 67.1% (47/70) of the pancreatic cancer tissues but in only 11.4% (8/70) of the adjacent non-tumor pancreatic tissues. Further, the difference in the positive rate of HIF-2α expression between the pancreatic cancer tissues and the non-tumor tissues was significant (χ2=45.549, *P*<0.05).

**Figure 2:**
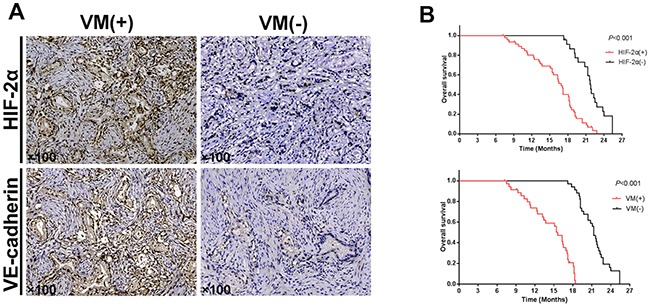
Correlation of HIF-2α, VE-cadherin, and VM in tissues, and the HIF-2α and VM expression survival curves in pancreatic cancer patients **(A)** Representative immunohistochemical staining of HIF-2α and VE-cadherin in the two groups of pancreatic cancer tissues exhibiting either VM-positive or VM-negative expression. Original magnification100×. The images are representative of three independent experiments. **(B)** The survival ratesof70 pancreatic cancer patients that were positive and negative for HIF-2α and VM expression (log-rank test, P<0.001).

As shown in Table 1, we also investigated the correlation of HIF-2α expression with clinicopathological features in the pancreatic cancer patients and found that HIF-2α expression was significantly correlated with tumor differentiation (χ2=6.921, *P*=0.026), clinical stage (χ2=6.460, *P*=0.017), and lymph node metastasis (χ2=5.250, *P*=0.040). However, the expression of HIF-2α had no obvious association with gender, age, tumor location, or tumor size (*P*>0.05). These results indicated that HIF-2α might be involved in poor differentiation and advanced clinical stages of pancreatic cancer. To assess the prognostic significance of HIF-2α expression on survival of pancreatic cancer patients further, a Kaplan-Meier analysis was conducted. The results indicated that patients with HIF-2α-negative expression had a significantly longer survival time than those with HIF-2α-positive expression (log-rank test, *P*<0.05; Figure 2B). In addition, the results of univariate analysis revealed that clinical stage (*P=*0.001), lymph node metastasis (*P=*0.001), and HIF-2α expression (*P=*0.001) were significantly associated with patient survival (Table 2). Finally, the results of the multivariate analysis revealed that clinical stage (*P=*0.002), lymph node metastasis (*P=*0.001), and HIF-2α expression (*P=*0.001) were independent and significant prognostic factors in pancreatic cancer patients (Table 3). In conclusion, the overexpression of HIF-2α was correlated with poor prognosis and was an independent prognostic marker of pancreatic cancer.

**Table 1:** Expression of HIF-2α and VM and the relation with the clinicopathologic features in pancreatic carcinoma

Variables	HIF-2α				VM		
	No.	Positive	Negative	*P*-value	Positive	Negative	*P*-value
Gender				0.306			0.473
Male	42	26	16		20	22	
Female	28	21	7		16	12	
Age (years)				1.000			0.467
≤ 65	41	28	13		23	18	
>65	29	19	10		13	16	
Tumor location				0.569			0.278
Head	52	36	16		29	23	
Body and tail	18	11	7		7	11	
Tumor size (cm)				0.122			0.147
≤2	27	15	12		17	10	
>2	43	32	11		19	24	
Differentiation				0.026			0.014
Well	8	3	5		2	6	
Moderate	17	9	8		5	12	
Poor	45	35	10		29	16	
Clinical stage				0.017			0.001
I	25	12	13		6	19	
II	45	35	10		30	15	
Lymph node metastasis							
Yes	38	30	8	0.040	25	13	0.016
No	32	17	15		11	21	

**Table 2:** Univariate analysis of survival time of pancreatic cancer patients (Kaplan-Meier)

Variables	Cases	Average survivalperiod (months)	P-value
Gender			0.571
Male	42	18.1±1.4	
Female	28	17.6±1.7	
Age (years)			0.668
≤ 65	41	18.0±1.3	
>65	29	17.6±1.7	
Tumor location			0.098
Head	52	17.3±1.2	
Body and tail	18	19.3±2.0	
Tumor size (cm)			0.858
≤2	27	18.0±1.7	
>2	43	17.8±1.3	
Differentiation			0.271
Well	8	21.8±1.2	
Moderate	17	21.7±1.4	
Poor	45	19.7±1.9	
Clinical stage			0.001
I	25	21.2±1.0	
II	45	15.9±1.2	
Lymph node metastasis			0.001
Yes	38	15.2±1.3	
No	32	20.8±0.8	
HIF-2α expression			0.001
Positive	47	16.0±1.2	
Negative	23	21.5±0.9	
VM expression			0.001
Positive	36	14.5±1.2	
Negative	34	21.2±0.7	

**Table 3:** Multivariate analysis of prognostic markers in pancreatic cancer patients

Variables	HR	95%CI	P-value
Gender	1.535	0.859-2.743	0.148
Age	1.456	0.815-2.602	0.205
Tumor location	0.622	0.329-1.177	0.144
Tumor size	0.690	0.398-1.197	0.186
Differentiation	1.023	0.679-1.541	0.914
Clinical stage	3.826	1.647-8.891	0.002
Lymph node metastasis	3.975	1.871-8.447	0.001
HIF-2α expression	4.694	2.033-10.837	0.001
VM expression	5.857	1.482-23.143	0.012

### Correlation between HIF-2α expression and VM in pancreatic cancer

To determine whether VM structures could be observed in tissues obtained from 70 pancreatic cancer patients, we utilized anti-CD34 and PAS staining to identify the endothelium and VM channels, respectively. CD34-positive staining identified vessels formed by endothelial cells (yellow arrow), whereas CD34-negative, PAS-positive vascular-like structures containing red blood cells formed by cancer cells or ECM were regarded as VM (red arrow; Figure 1B). We found that 51.4% (36/70) of the tissues contained VM channels within the pancreatic cancer tissues; however, none of the tissues in the non-tumor tissue group exhibited a VM pattern. Further, we found that the expression of VM channels was correlated with tumor differentiation (χ2=8.588, *P*=0.014), clinical stage (χ2=11.712, *P*=0.001), and lymph node metastasis (χ2=6.863, *P*=0.016), whereas the expression of VM had no obvious association with gender, age, tumor location, or tumor size (*P*>0.05). As well, the patients in the VM-positive group had a poorer prognosis than those in the VM-negative group (log-rank test, *P*<0.05; Figure 2B). The univariate analysis results indicated that patients with VM expression had a high risk of reduced survival time (*P=*0.001, Table 2). In addition, the results of the multivariate analysis using Cox's proportional hazards model revealed that VM expression was also an independent and important prognostic factor in all patients (*P=*0.012, Table 3). These results indicated that VM is associated with the prognosis of pancreatic cancer patients.

To investigate the relationship betweenHIF-2α expression and VM in 70 pancreatic cancer tissues, we examined the expression of HIF-2α and VE-cadherin in both the VM-positive and VM-negative groups. As the most critical protein for VM, VE-cadherin expression was significantly higher in the VM-positive group (25/36) than in the VM-negative group (13/34; χ2=6.044, *P*<0.05). Further, HIF-2α expression was detected in 80.6% (29/36) of the VM-positive group but in only 52.9% (18/34) of the VM-negative group. As well, the difference in the positive rate of HIF-2α expression between the VM-positive and VM-negative groups was significant (χ2=6.863, *P*<0.05; Figure 2A). In addition, there was a significant positive correlation between the expression of HIF-2α and VM (r=0.294, *P*<0.05) and between the expression of VE-cadherin and VM (r=0.313, *P*<0.05) in the pancreatic cancer tissues. We performed stratifed analysis of survival time between HIF-2α^+^VM^+^ group and HIF-2α^+^VM^-^ group. The results indicated that patients with HIF-2α^+^VM^+^ group had a longer survival time than those with HIF-2α^+^VM^-^ group (log-rank test, P<0.05, Supplementary Figure 1). The result indicated that HIF-2α positive group with VM expression had a high risk of reduced survival time.

### HIF-2α promotes the migration, invasion, and formation of VM in pancreatic cancer *in vitro*

**Figure 3:**
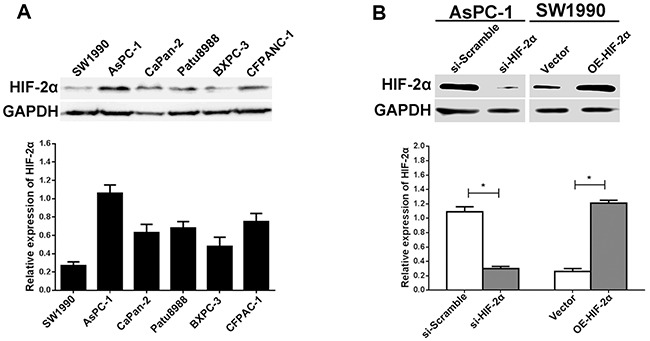
Expression of HIF-2α in pancreatic cell lines and regulation of HIF-2α in AsPC-1 and SW1990 cells **(A)** The relative expression of HIF-2α in pancreatic cancer cell lines (SW1990, AsPC-1, CaPan-2, PaTu8988, BXPC-3, and CFPANC-1) was measured by Western blot. **(B)** Knockdown and ectopic expression of HIF-2α in AsPC-1 and SW1990 cells by Western blot. The images are representative of three independent experiments. * = *P*<0.05.

To validate the expression of HIF-2α in pancreatic cancer cells, we performed western blotting using the SW1990, AsPC-1, CaPan-2, Patu8988, BxPc-3, and CFPANC-1 pancreatic cancer cell lines. Based on the results, we determined that AsPC-1 cells had the highest HIF-2α expression, in contrast with the SW1990 cells, which expressed the lowest level (Figure 3A). To investigate the effect of HIF-2α on VM, we selected AsPC-1 cells to conduct the HIF-2α silencing experiments by siRNA, whereas SW1990 cells were transfected with HIF-2α cDNA to upregulate HIF-2α expression. As shown in Figure 3B, the expression of HIF-2α was obviously decreased in AsPC-1 cells after si-HIF-2αtreatment (*P*<0.05), whereas the expression of HIF-2α was significantly upregulated in SW1990 cells following transfection with OE-HIF-2α (*P*<0.05).

**Figure 4:**
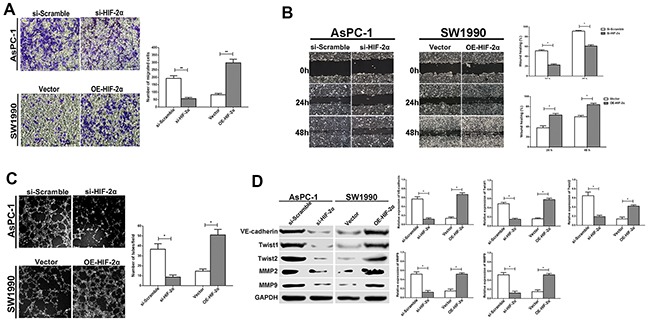
HIF-2α promotes cell migration, invasion, and VM formation *in vitro* **(A)** Cell invasion was detected using a Transwell® assay. Representative images of cell invasion captured under an inverted microscope (original magnification, 200×). The data represent the means ± SD of five experiments. ** = *P*<0.01. **(B)** Cell migration was detected using a wound scrape assay. Representative images of cell migration in the wound scrape model at 0, 24, and 48 h are shown; original magnification, 100×. The data represent the means ± SD of three experiments. * = *P*<0.05. **(C)** Changes in VM formation in three-dimensional culture *in vitro*. Representative images captured under an inverted microscope (original magnification, 200×). **(D)** The effect of HIF-2α on expression of VM-related proteins (VE-cadherin, Twist1, Twist2, MMP2, and MMP9) was examined by Western blot. The images are representative of three independent experiments. * = *P*<0.05

Because VM is closely related to invasion and migration in tumor cells, we measured invasion ability and cell migration using the Transwell® system and a wound healing assay after performing HIF-2α knockdown or HIF-2α ectopic transfection, respectively. We found that the si-HIF-2α pancreatic cells with knocked-down HIF-2α exhibited decreased tumor cell invasion compared with the si-Scramble group (*P*<0.01). However, the upregulation of HIF-2α expression in the OE-HIF-2α group promoted cell invasion compared with the vector group (*P*<0.01; Figure 4A). A similar trend was observed in the wound healing assay, as the si-HIF-2α cells displayed relatively slower migration towards the wound space compared with the si-Scramble cells; however, upregulation of HIF-2α expression could enhance the migration speed toward the wound space (*P*<0.05; Figure 4B).

To confirm the role of HIF-2α in VM formation, 3D tumor cell culture was used to observe VM formation *in vitro*. Notably, the number of complete tubular channels formed by AsPC-1 cells was significantly decreased in the si-HIF-2α cells compared with si-Scramble cells in 3D cultures (*P*<0.05). In contrast, the overexpression of HIF-2α in SW1990 cells promoted the formation of more typical ECM-rich vessel-like networks by tumor cells in the OE-HIF-2α group compared with the vector group (*P*<0.05; Figure 4C). These data indicated that HIF-2α promoted the formation of VM in pancreatic cancer cells *in vitro*.

Next, we examined the levels of the VM related marker VE-cadherin, as well as Twist1, Twist2, MMP2, and MMP9, which are important to cell plasticity and VM formation, in cells exhibiting altered HIF-2α expression via si-RNA or overexpression. As shown in Figure 4D, the expression of VE-cadherin, Twist1, Twist2, MMP2, and MMP9 were significantly decreased in si-HIF-2α AsPC-1 cells compared with si-Scramble cells (*P*<0.05). Similarly, after upregulating HIF-2α by transfecting HIF-2α cDNA, the expression of VM-associated proteins including VE-cadherin, Twist1, Twist2, MMP2, and MMP9 were all increased in the OE-HIF-2α group compared with the negative vector group in the SW1990 cells(*P*<0.05). These results confirmed our hypothesis that HIF-2α enhanced VM-like channel formation, which might be involved in regulating VM-related proteins.

### HIF-2α promotes VM formation through Twist1 binding to VE-cadherin in pancreatic cancer

**Figure 5:**
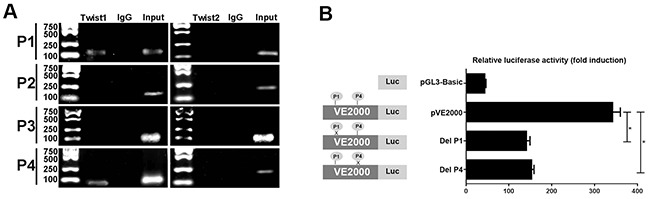
The transcriptional factor of Twist1 binding to the promoter region of VE-cadherin **(A)** ChIP assay of Twist1 and Twist2 interactions with the VE-cadherin promoter. Bands are PCR products targeting P1-P4 of the VE-cadherin promoter. Specific anti-Twist1, anti-Twist2, or control normal mouse IgG was used for immunoprecipitations, whereas genomic DNA was used as the input control. **(B)** The effect of Twist1 on the transcriptional activity of the VE-cadherin promoter. Mutated promoters that specifically delete P1 or P4 of the Twist1 binding elements were introduced into AsPC-1 cells. Firefly luciferase expression levels were normalized to the luciferase activity of the internal Renilla control. The results indicate that both P1 and P4 elements are positive regulatory elements. Separate experiments performed in triplicate for each group. One-way analysis of variance (ANOVA) was used, * = *P*<0.05.

Twist is reportedly a main transcription factor associated with the EMT progress [23]. First, we used Patch software to identify potential transcription factor binding sites in the promoter region of the VE-cadherin gene. Among these transcription factors, we found that Twist was important. Four potential Twist protein binding sites, separately designated P1 (-421bp), P2 (-714bp), P3 (-2000bp), and P4 (-2110bp) were identified in the Patch transcription factor binding site database (Table 4). Next, we performed a ChIP assay using AsPC-1 cells overexpressing Twist1 and Twist2 to investigate whether Twist1 and Twist2 could bind to the promoter region of VE-cadherin. Based on the results presented in Figure 5A, we found that the Twist1 antibody only specifically immunoprecipitated a Twist1-DNA complex in the P1 and P4 regions of the VE-cadherin promoter, and that Twist1 had no positive binding capacity to the other two VE-cadherin binding sites. However, there was no significant evidence to illustrate that Twist2 had the capacity to bind to the four potential transcription regions of VE-cadherin via the ChIP assay. The results suggested that Twist1 directly bound to the VE-cadherin promoter through the P1 and P4 regions.

**Table 4:** Predict potential Twist binding sites in the promotor region of VE-cadherin gene (Patch software)

Twist binding site	Position	Sequence
P1	-417bp to -431bp	gtca**CACGTG**actcc
P2	-660bp to -674bp	tccc**CAAATG**tcaga
P3	-1996bp to -2014bp	cagc**CAGGCCTCCC**tcgcc
P4	-2106bp to -2124bp	ggaa**CAGAAACATC**cctca

To understand the effect of the P1 and P4 elements on VE-cadherin transcription, we constructed recombinants containing wild type and mutated promoters of VE-cadherin and conducted luciferase reporter gene assays to evaluate their effects. The normal pVE2000 luciferase promoter construct displayed increased transcription activity; however, the deletion of P1 or P4 in the promoter caused an obvious decrease in transcription activity (Figure 5B). Our results suggest that the P1 and P4 Twist1 binding sequence regions are positive regulatory elements for VE-cadherin transcription.

**Figure 6:**
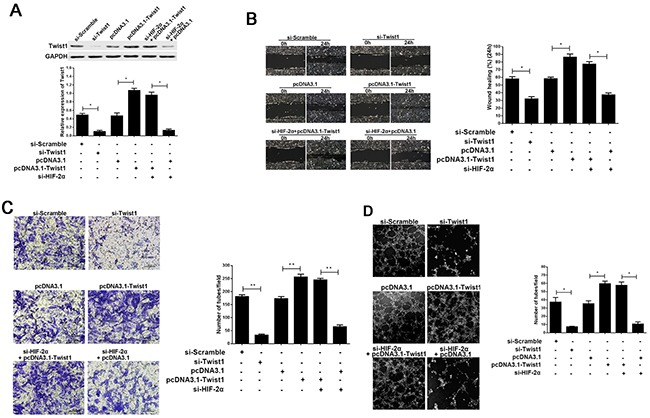
The effect of Twist1 in the progress of HIF-2α promoting cell migration, invasion, and VM formation *in vitro* **(A)** Expression of Twist1 after regulating HIF-2α or Twist1 by Western blot. The images are representative of three independent experiments. * = *P*<0.05. **(B)** Cell invasion was detected using a Transwell® assay. Representative images of cell invasion captured under an inverted microscope (original magnification, 200×). The data represent the means ± SD of five experiments. ** = *P*<0.01. **(C)** Cell migration was detected using a wound scrape assay. Representative images of cell migration in the wound scrape model at 0, 24 h are shown; original magnification, 100×. The data represent the means ± SD of three experiments. * = *P*<0.05. **(D)** Change in VM formation after regulating HIF-2α or Twist1 by three-dimensional cell culture *in vitro*. The images are representative of three independent experiments (original magnification, 200×). * = *P*<0.05.

To confirm that Twist1 is involved in HIF-2α-induced VM channel formation, we first silenced Twist1 expression by RNAi and then upregulated Twist1 expression by pcDNA3.1-Twist1 (P<0.05, respectively; Figure 6A). Figure 6B, 6C and 6D indicated that Twist1, a main VM-related proteins, promotes the migration, invasion and VM formation in pancreatic cancer cells; While downregulating Twist1 in AsPC-1 cells had the opposite effect (P<0.05, respectively). Next, we examined whether the restoration of Twist1 could rescue VM formation after HIF-2α silencing. The co-transfection of pcDNA3.1-Twist1 and si-HIF-2α in AsPC-1 cells efficiently restored Twist1 expression, and partially reversed the suppression of VM formation induced by HIF-2α silencing (P<0.05, respectively; Figure 6D). These results indicated that Twist1 could contribute to VM formation, and that Twist1 could also, at least partly, revert the ability of HIF-2α to induce VM formation in pancreatic cancer cells. These findings indicated that HIF-2α might regulate Twist1 binding to VE-cadherin to promote VM formation in pancreatic cancer cells.

### HIF-2α promotes the expression of Twist1 and VE-cadherin and contributes to VM formation in pancreatic cancer *in vivo*

**Figure 7:**
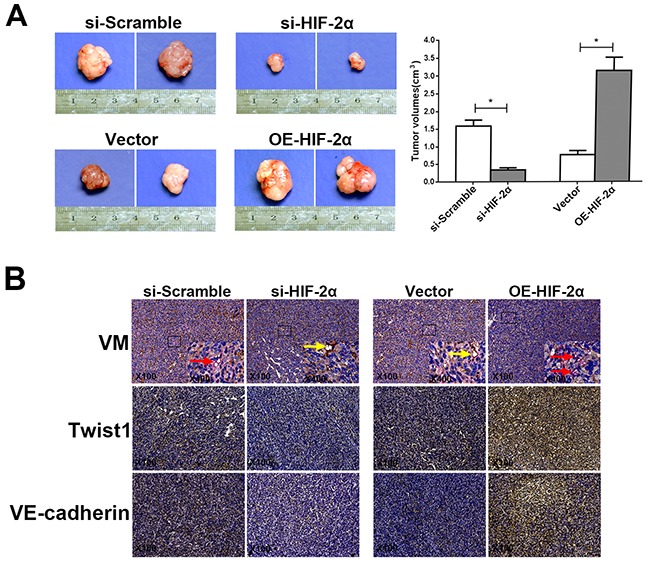
HIF-2α promotes VM formation *in vivo* **(A)** Size and tumor volume of the pancreatic cancer xenografts harvested two weeks after orthotopic inoculation. * = *P*<0.05. **(B)** Representative CD34/PAS staining (Red arrows indicate typical VM structures, whereas yellow arrows indicate endothelial vessels). Images of Twist1 and VE-cadherin expression in tumor sections from pancreatic cancer xenografts with silencing or overexpression of HIF-2α and their corresponding controls. Original magnification100× or 400×. The images are representative of three independent experiments

We reported that HIF-2α can promote VM formation *in vitro*. To investigate whether HIF-2α contributes to VM *in vivo*, we established xenografts in nude mice and then transplanted AsPC-1 cells with stably silenced HIF-2α and SW1990 cells that stably overexpressed HIF-2α, as well as the respective empty vectors, into the pancreas of each mouse. After two weeks, we observed the tumor volume and investigated the expression of VM, Twist1, and VE-cadherin in the murine pancreatic tumor tissues. As shown in Figure 7A, the tumors were significantly smaller in the si-HIF-2α group than in the si-Scramble group (P<0.05), whereas the tumors in the OE-HIF-2α group were larger compared with the vector group (P<0.05). CD34/PAS staining revealed that VM was obviously reduced in the si-HIF-2α xenografts compared with the si-Scramble group (P<0.05). In contrast, VM was markedly increased in the OE-HIF-2α xenografts compared with the vector group (P<0.05). Twist1 and VE-cadherin, which are vital VM proteins, were more highly expressed in the OE-HIF-2α xenografts compared with the vector group (*P*<0.05); however, the expression of both Twist1 and VE-cadherin was lower in the si-HIF-2α group compared with the matched group (*P*<0.05; Figure 7B).

## DISCUSSION

An increasing number of studies have indicated that tumor angiogenesis not only includes endothelium-dependent vessels, but is also dependent on VM formed by cancer cells and ECM [24]. In fact, VM channels, mosaic blood vessels, and endothelial vessels coexist in malignant tumors and can exchange the stage of each other when the tumor microenvironment changes [25]. Following the progression of pancreatic cancer, tumors require additional perfusion pathways to obtain sufficient oxygen and nutrients from the blood. As a new and unusual source of blood supply to solid tumors, VM is clinically significant in pancreatic cancer and is associated with poor prognosis [20, 26]. In our study, we observed VM channel patterns in pancreatic cancer tissues, and demonstrated that VM channels were correlated with poor tumor differentiation, advanced clinical stage, and lymph node metastasis. In addition, VM-positive expression was associated with shorter overall survival than VM-negative expression in pancreatic cancer patients.

Growing solid tumors reach hypoxia because the blood supply in their normal development state is lacking, which induces the expression of HIF-1α and HIF-2α and contributes to the growth of the tumor in this condition [27]. In the present study, we discovered that the expression of HIF-2α was high in human pancreatic cancer tissues, and that the increased expression of HIF-2α caused clinicopathological disadvantages in pancreatic cancer patients. Hypoxia can stimulate the formation of tumor vessel structures including VM and angiogenesis, which further provides a convenient environment for tumor invasion and metastasis [28]. Our study indicated that HIF-2α could promote invasion and cell migration in pancreatic cancer cell lines *in vitro*. We also found that HIF-2α enhanced the ability of pancreatic cancer cells to form VM structures both *in vitro* and *in vivo*. Based on our results, it appears that HIF-2α not only plays a vital role in tumor cell invasion and migration but also closely associated with tumor cell plasticity in VM patterns.

To elucidate the role of HIF-2α in promoting VM formation, additional experiments were conducted. Tumor cells involved in VM formation exhibit a high degree of plasticity and an obvious multipotent phenotype that is similar to embryonic stem cells [29]. In malignant tumors, unlike normal embryonic progenitors, plastic tumor cells lack vital regulatory checkpoints, which stimulates the aberrant activation of embryonic signaling pathways and leads to unregulated growth and aggressive behavior in these cells [30]. As a member of the cadherin family of transmembrane proteins, VE-cadherin is an adhesive protein expressed specifically in endothelial cells that accelerates homotypic cell-to-cell interactions and is involved in vasculogenic activities [17, 31]. Highly aggressive tumor cells assume the endothelial cell phenotype to mimic the structure of the vessels. Further, VE-cadherin is an important vascular-associated gene that participates in VM formation. VE-cadherin is highly expressed in aggressive melanoma cells, and the downregulation of VE-cadherin expression inhibits VM formation [32]. Additionally, studies have reported that MMP2 and MMP9 also participate in VM formation by regulating the PI3K/Akt signal pathway [33]. In this study, the expression of HIF-2α and VE-cadherin was higher in the VM-positive group than in the VM-negative group. We likewise discovered that the expression of VE-cadherin, MMP2, and MMP9 was also upregulated following the upregulation of HIF-2α and that the downregulation of HIF-2α resulted in decreased VE-cadherin, MMP2, and MMP9 expression. Thus, it seems reasonable to conclude that HIF-2α is involved in VM formation through the regulation of several important VM-associated proteins, especially VE-cadherin.

The transcription factor Twist, a master regulator of embryonic morphogenesis, is closely related to many kinds of cancers and promotes metastasis of tumor cells by contributing to VM formation. As a main VM-related proteins, twist is a member of the basic helix-loop-helix (bHLH) transcription factor family and structural similarity with HIF at the bHLH [20]. The function of HIF and Twist may have some similarity. So, we focused on Twist protein including Twist1 and Twist2. Twist1 and Twist2 belong to the bHLH family of transcription factors, and they share high sequence homology and structural similarity [34, 35]. In our study, the ChIP assay results showed that Twist1, but not Twist2, could promote VE-cadherin transcription in the -421 bp and -2110 bp promoter region sites of the VE-cadherin gene. As well, the results of the luciferase reporter gene assays indicated that the -421 bp and -2110 bp regions of the Twist1 binding sequences are positive regulatory elements for VE-cadherin transcription and can increase the activity of VE-cadherin. However, the role of Twist2 in VM remains unclear. As important transcription factors in VM formation, the expression of Twist1 was decreased after HIF-2α was downregulated in AsPC-1 cells, whereas the opposite results were observed after upregulating HIF-2α in SW1990 cells *in vitro* and *in vivo*. Furthermore, restoring the expression of Twist1 rescued the ability of HIF-2α to form VM structures. These results indicated that Twist1 could promote VM formation, and that HIF-2α promoted VM formation through the binding of Twist1 to the VE-cadherin promoter.

VM, a novel and angiogenesis-independent pathway, was considered as a challenge to traditional anti-angiogenic treatments and poor prognosis in tumor patients. In the past, potential therapies aimed at endothelial cells have gained much attention [36]. However, the effects of anti-angiogenesis drugs have been unsatisfactory in these malignant tumors that have other blood supply mechanisms such as VM. Furthermore, when endothelial vessels are blocked or the blood vessel density is reduced due to anti-angiogenic agents, the tumor might reach hypoxia. Subsequently, the oxygen and nutrient deficiencies contribute to VM formation and indirectly promote tumor progression. Therefore, strategies that combine traditional endothelium-dependent drugs with VM-targeted therapies have garnered much attention and interest. Recently, many studies have focused on various mechanisms and genes involved in VM formation, such as VE-cadherin and MMPs. The development of an anti-VM therapy should consider several important aspects including suppressing the plasticity of tumor cells, cutting off the molecular signaling pathways of VM formation, and the remodeling of the tumor microenvironment [37, 38]. However, VM and anti-VM are still in the initial stages and additional studies are required to elucidate the specific mechanisms of VM. Nonetheless, whether blockage of HIF-2α could be an efficient way to inhibit VM formation deserves further exploration.

In conclusion, our study demonstrated that HIF-2α and VM were overexpressed in pancreatic cancer tissues and were associated with poor pathological characteristics. HIF-2α contributes to VM formation by regulating the expression of VE-cadherin through the binding of the transcription factor Twist1 to the promoter of VE-cadherin in pancreatic cancer both *in vitro* and *in vivo*. Thus, VM therapies should focus on HIF-2α or other VM-associated genes to find more effective ways to treat pancreatic cancer.

## MATERIALS AND METHODS

### Clinical samples

Tumor tissues were obtained from 70 patients at the First Affiliated Hospital of Soochow University from January 2011 to December 2013, including tumor samples and matching adjacent non-tumor tissues. Detailed clinicopathological data were recorded, including the age and gender of each patient, and the tumor size, tumor differentiation, and lymph node metastasis. The tumor clinical stages were classified according to the Union for International Cancer Control (UICC) staging system. None of the patients received chemotherapy, radiotherapy, or immunotherapy prior to surgery. All samples were obtained following patient consent and approval by the Ethics Committee of Soochow University.

### Cell culture

The AsPC-1, CaPan-2, PaTu8988, SW1990, and BXPC-3 pancreatic cancer cell lines were obtained from the Chinese Academy of Sciences (Shanghai, China). The cells were maintained in Dulbecco's Modified Eagle Medium (DMEM; HyClone, Shanghai, China) supplemented with 10% fetal bovine serum (FBS; HyClone, Shanghai, China) and cultured at 37°C in a humidified atmosphere containing 5% CO2.

### Lentiviral vector production and cell infection

Lentiviral vector system was purchased from GeneChem (Shanghai, China). This vector system include three plasmids: GV115 or GV358, pHelper 1.0 and pHelper 2.0. According to the manufacturer's instruction, lentiviral constructs GV115 containing the siRNA sequence targeting human HIF-2α (5’-GCAAATGTACCCAATGATA-3’) was used to establish cell lines constitutively repressing HIF-2α (named si-HIF-2α). A nonspecific scrambled siRNA sequence (sequence: 5’- GTTCTCCGAACGTGTCACGT-3’) was used as a negative control (si-Scramble). Full-length HIF-2α was synthesized and subcloned into a GV358 vector. Lentiviral vector encoding the human HIF-2α gene was designated OE-HIF-2α. The empty vectors served as a negative control (Vector). Pancreatic cancer cell lines were grown to 30-40% confluence before lentiviral infection. The viral supernatant was added at a multiplicity of infection (MOI) of 5 per well with 5 ug/ml Polybrene. Cells were cultured in normal growth medium for 72 h after infection and expanded for downstream experiments.

### Cell transfection

Twist1 cDNA was cloned into the mammllian expression vector pcDNA3.1 (Invitrogen, USA). SiRNA sequence targeting human Twist1 (5’- GCAAGAUU

CAGACCCUCAATT-3’) was designed and synthesized by GeneChem (Shanghai, China). To investigate the correlation between HIF-2α and Twist1 expression, AsPC-1 cells expressing either pcDNA3.1 or pcDNA3.1-Twist1 were transfected with the GV115-based construct containing HIF-2α shRNA. Vector and siRNA transfections were performed using Lipofectamine 2000 (Invitrogen, USA) according to the manufacturer's protocol. Cells were incubated for 24 h before use in experiments.

### Three-dimensional (3D) culture

A 24-well culture plate was coated with 250 μl/well of growth factor-reduced Matrigel™ (BD Biosciences), which was allowed to polymerize for 1 hour at 37°C. A cell suspension (5 × 10^5^ cells/well) was seeded on top of the gel, and three wells were used for each group of AsPC-1 cells and SW1990 cells. After adding DMEM (HyClone, Shanghai, China) containing 10% FBS (HyClone, Shanghai, China), the cells in the 3D culture system were observed at 12 and 24 hours for the number and the completeness of the tubules. Images were captured using an inverted light microscope at 200× magnification (Leica Microsystems, Mannheim, Germany) after incubation for 24 hours.

### Western blot

The cells were collected and lysed on ice in RIPA lysis buffer (Beyotime, Shanghai, China). Total protein extracts were separated by 10% SDS-PAGE and transferred to PVDF membranes. The membranes were blocked with 10% non-fat milk powder at room temperature for 2 hours and incubated with anti-HIF2α (ab8365, 1:200), anti-VE-cadherin (ab33168, 1:1000), anti-MMP2 (ab37150, 1:1000), anti-MMP9 (ab76003, 1:5000), anti-Twist1 (ab50887, 1:200), anti-Twist2 (ab66031, 1:50; all from Abcam, Cambridge, UK), and anti-GAPDH (WL01743, 1:1000, Santa Cruz Biotechnology, CA, USA) primary antibodies at 4°C overnight. After three washes, the membranes were incubated with a horseradish peroxidase-conjugated goat anti-mouse IgG (250137, 1:2000; Santa Cruz Biotechnology). Reactive bands were detected using an enhanced chemiluminescence western blotting detection reagent (GE Healthcare, USA).

### Wound healing assay

Cells from each group were plated in 6-well plates. When the cells reached 80% confluence, a small wound area was created in the confluent monolayer by making a lengthwise swipe using a 200-μl pipette tip. The cells were washed twice with PBS and incubated at 37°C. The speed of wound closure was measured by calculating the ratio of the distance of the wound closure at 24 and 48 hours compared to 0 hours, respectively. The wound width was measured at 100× magnification using a microscope (Leica Microsystems, Mannheim, Germany). Each experiment was performed in triplicate.

### Cell invasion assay

*In vitro* cell invasion assays were performed using Transwell® cell culture chambers with 8 μm pores (Corning, NY, USA). The inserts in the membrane filter were coated with 40ul configured Matrigel™ on the upper surface. The cells were resuspended in serum-free DMEM at a concentration of 5 × 10^5^ cells/ml and placed in the upper chamber. The lower chamber was filled with DMEM with 10% FBS. After incubation at 37°C for 48 hours, the cells on the upper surface of the filter were removed with a cotton swab. The invading cells at the bottom of the Matrigel™ were fixed in methanol and stained with 0.1% crystal violet. The number of invading cells in five random fields per well was calculated using a microscope at 200× magnification. Each assay was performed in triplicate.

### Chromatin immunoprecipitation assay

A chromatin immunoprecipitation (ChIP) assay was performed using a ChIP assay kit (Upstate Biotechnology, LP, USA) as described by the manufacturer. The AsPC-1 cells were lysed and the immunoprecipitation was performed using anti-Twist1 polyclonal antibody (Santa Cruz Biotechnology, CA, USA), anti-Twist2 monoclonal antibody (Abcam, Cambridge, UK), or mouse immunoglobulin G (IgG; negative control). After washing, the antibody-protein-DNA complex was eluted from the beads and reversed cross-link incubation. After removing protein and RNA, the purified DNA was subjected to polymerase chain reaction (PCR) using primers specific for the human VE-cadherin promoter. The PCR primers were all designed by GeneChem (Shanghai, China) as follows: P1-F: 5′-CTGCTCCCATTCACTGTA

AGAC-3′ and P1-R: 5′-AGACAGATTGGAGGG GCTAG-3′ (103bp); P2-F: 5′-TCCTGGCATTCCTCC TTCA-3′ and P2-R: 5′-CCTGGAGTCGAGGTTTGGA

-3′ (148bp); P3-F: 5′-AGCCAGCCCAGCCCTC AC-3′ and P3-R: 5′-CCTGTCA

GCCGACCGTCTTTG-3′ (149bp); P4-F: 5′-AGCCC TCACAAAGGAACAAT-3′ and P4R: 5′-CTTCCCA GGAGGAACAGATC -3′ (241bp).

### Luciferase reporter assay

The VE-cadherin promoter regions (approximately 2 kb surrounding the transcription start site) were generated by PCR amplification of genomic DNA and cloned into the pGL3-basic promoter vector (Promega). The sequence of mutated P1 is 5’-CACGTG-3’ and the sequence of mutated P4 is 5’-CAGAAAAATC-3’. Luciferase reporter assays were performed by transfecting the mutated VE-cadherin promoter reporter plasmid (separate deletions of P1 or P4 in the promoter), together with the basic pRL vector, into AsPC-1 cells (Shanghai, China) in triplicate using Lipofectamine® 2000 (Invitrogen). The cell lysates were analyzed for luciferase activity using the Dual-Luciferase® Assay Kit according to the manufacturer's instructions. Each experiment was performed in triplicate.

### Immunohistochemical and CD34/ PAS double-staining

The serial sections (4 μm) subjected to immuno-histochemical(IHC) staining were fixed in freshly prepared 3% H_2_O_2_ with 0.1% sodium azide to quench endogenous peroxidase and then treated with antigen retrieval solution for 15 min. After placing in blocking reagent for 15 min, the sections were incubated in primary anti-HIF-2α (1:500, Abcam), anti-VE-cadherin (1:500, Abcam), anti-Twist1 (1:500, Abcam), and anti-CD34 (1:200, Abcam) monoclonal antibody overnight at 4°C, followed by incubation with the secondary antibody and ExtrAvidin®-conjugated horseradish peroxidase. After IHC staining for CD34, the sections were washed with running water for 5 min and incubated with PAS stain for 15 min.

The staining intensity was scored as follows: 0, no staining; 1, weak staining; and 2, moderate to strong staining. The percentage of positively stained cells was scored as follows: 0, <10%; 1, 10%–50%; and 2, >50%. The final score was calculated as the sum of the intensity and quantity scores. A score >2 indicated positive expression. CD34 staining was performed to identify endothelial cells, and any structure containing CD34-positive immunoreactivity was defined as a blood vessel. PAS staining was used to identify matrix-associated vascular channels in the pancreatic cancer tissues. Vessels lined by endothelial cells, regardless of the presence of a basement membrane, were counted as endothelium-dependent vessels. In contrast, VM structures were defined as CD34-negative, PAS-positive structures.

### Xenografts in nude mice

Twenty 6-week-old nude female mice were randomized into four groups (si-Scramble, si-HIF-2α, vector, and OE-HIF-2α, respectively). All protocols used in the animal experiments were approved by the institutional animal ethics committee. To evaluate tumor formation, 3 × 10^6^ cells/0.2 mL were suspended and injected into the pancreas of each nude mouse. Tumor size was determined by caliper measurements of the length and width. Tumor volume was calculated using the formula tumor volume = (length × width)^2^/2. Two weeks after injection, the mice were sacrificed and the tumors were harvested, fixed with formalin, and embedded in paraffin.

### Statistical analysis

All data in the study were evaluated using SPSS version 18.0 software. All data were presented as mean ± SD. The continuous variables were compared using one-way analysis of variance (ANOVA) and categorical variables were compared using a Chi-square test. The correlation analysis was performed using a Spearman's Rank Correlation test. Survival was assessed according to the Kaplan-Meier method and compared using the log-rank test. The multivariate analysis of prognostic markers was performed using the Cox proportional hazards regression model. Differences were considered significant at values of P<0.05.

## SUPPLEMENTARY MATERIALS FIGURE



## Abbreviations

HIF-2α: is short for hypoxia-inducible factor-2α
VM: is short for vasculogenic mimicry
EMT: is short for Epithelial-mesenchymal transition
IHC: is short for immunohistochemistry
ChIP: is short for Chromatin immunoprecipitation
ECM: is short forextracellular matrix.
