# Supplementary material for: HIF-2α promotes the formation of vasculogenic mimicry in pancreatic cancer by regulating the binding of Twist1 to the VE-cadherin promoter
Source: Oncotarget. 2017 May 18;8(29):47801–15. doi: 10.18632/oncotarget.17999 (PMC5564606; doi:10.18632/oncotarget.17999)
Supplement: Supplementary file 1 [file oncotarget-08-47801-s001.pdf]

HIF-2α promotes the formation of vasculogenic mimicry in pancreatic cancer by regulating the binding of Twist1 to the VE-cadherin promoter

SUPPLEMENTARY MATERIALS

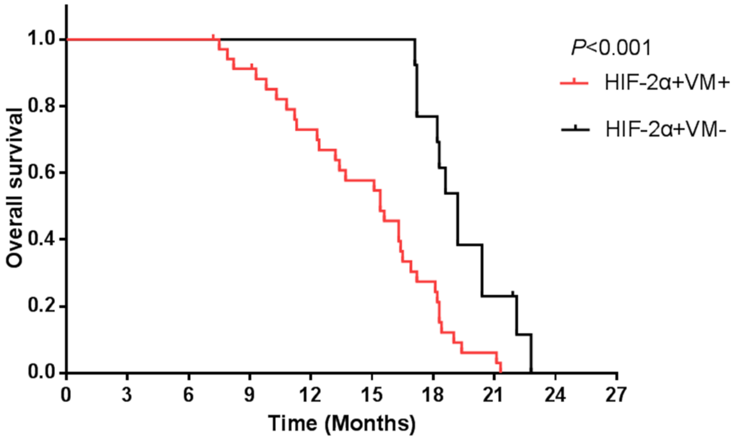

Supplementary Figure 1: Survival curves of HIF-2α+VM+ group and HIF-2α+VM- group in pancreatic cancer patients (log-rank test, P<0.05).
